# SHP-2-Induced Activation of c-Myc Is Involved in PDGF-B-Regulated Cell Proliferation and Angiogenesis in RMECs

**DOI:** 10.3389/fphys.2020.555006

**Published:** 2020-11-23

**Authors:** Jun Ma, Wenyi Tang, Ruiping Gu, Fangyuan Hu, Lei Zhang, Jihong Wu, Gezhi Xu

**Affiliations:** ^1^Eye Institute, Eye & ENT Hospital, Shanghai Medical College, Fudan University, Shanghai, China; ^2^Shanghai Key Laboratory of Visual Impairment and Restoration, Fudan University, Shanghai, China; ^3^Department of Ophthalmology, Eye and ENT Hospital of Fudan University, Shanghai, China; ^4^Department of Radiation Oncology, Renji Hospital, School of Medicine, Shanghai Jiao Tong University, Shanghai, China

**Keywords:** retina, proliferation, angiogenesis, PDGF-B, SHP-2

## Abstract

**Background**: Aberrant neovascularization resulting from inappropriate angiogenic signaling is closely related to many diseases, such as cancer, cardiovascular disease, and proliferative retinopathy. Although some factors involved in regulating pathogenic angiogenesis have been identified, the molecular mechanisms of proliferative retinopathy remain largely unknown. In the present study, we determined the role of platelet-derived growth factor-B (PDGF-B), one of the HIF-1-responsive gene products, in cell proliferation and angiogenesis in retinal microvascular endothelial cells (RMECs) and explored its regulatory mechanism.

**Methods**: Cell counting kit-8 (CCK-8), bromodeoxyuridine (BrdU) incorporation, tube formation, cell migration, and Western blot assays were used in our study.

**Results**: Our results showed that PDGF-B promoted cell proliferation and angiogenesis by increasing the activity of Src homology 2 domain-containing tyrosine phosphatase 2 (SHP-2) in RMECs, which was attenuated by the inhibition of PDGF receptor (PDGFR) or SHP-2 knockdown. Moreover, activation of c-Myc was involved in the processes of PDGF-B/SHP-2-driven cell proliferation in RMECs. The promoting effects of PDGF-B/SHP-2 on c-Myc expression were mediated by the Erk pathway.

**Conclusion**: These results indicate that PDGF-B facilitates cell proliferation and angiogenesis, at least in part, *via* the SHP-2/Erk/c-Myc pathway in RMECs, implying new potential treatment candidates for retinal microangiopathy.

## Introduction

Neovascularization is a multistep process, and a variety of growth factors are involved in regulating this process (Yang et al., 2016). Retinal angiogenesis, one of the major causes of visual loss in the world, is closely related to diseases such as diabetic retinopathy, retinopathy of prematurity, and age-related macular degeneration (Das and McGuire, 2003). Abnormal retinal neovasculatures, accompanied by the increased vascular permeability and functional incompetence, are prone to intraocular vascular leakage and bleeding, which leads to the development and progression of macular edema formation, retinal and vitreous bleeding, tractional retinal detachments, and ultimately irreversible visual loss (Miller et al., 2013). Although clinical intravitreal injections of anti-vascular endothelial growth factor (VEGF) therapy are widely utilized for the treatment of retinal angiogenesis, a significant portion of patients do not obviously benefit, and some patients become refractory to the therapy over time (Forooghian et al., 2009). Therefore, it is necessary to study the molecular mechanism regulating retinal angiogenesis.

The platelet-derived growth factor (PDGF) family includes four homodimers (PDGF-AA, PDGF-BB, PDGF-CC, and PDGF-DD) and one heterodimer (PDGF-AB). PDGFs achieve diverse functions by binding with their corresponding PDGF receptors (PDGFRs; Reigstad et al., 2005). There are two homodimers of the PDGFRs (PDGFR-αα and PDGFR-ββ). PDGF-AA, -AB, -BB, and -CC can activate PDGF receptor-α (PDGFRα), while PDGFRβ is activated by either PDGF-BB or -DD (Kazlauskas, 2017). The roles of PDGF proteins in regulating mitogens and angiogenesis have been studied in pulmonary hypertension (Grimminger and Schermuly, 2010) and several types of cancers (Hosaka et al., 2013; Crino and Metro, 2014). Furthermore, accumulating evidence has indicated the critical roles of PDGF-B, one of the HIF-1-responsive gene products, in the retina. PDGF-B is essential for the maintenance of the retinal vasculature (Lindahl et al., 1997). Increased expression of PDGF-B and its receptors is observed in the many proliferative retinal membranes (Robbins et al., 1994). Severe neovascularization is induced by retina-specific expression of PDGF-B in mice (Seo et al., 2000). Treatment with high glucose in human capillary endothelial cells leads to the activation of the PDGF-B/PDGFR pathway (Inaba et al., 1996). However, the mechanism of PDGF-B-regulated retinal angiogenesis remains to be elucidated.

Src homology 2 domain-containing tyrosine phosphatase 2 (SHP-2), encoded by *PTPN11*, is ubiquitously expressed as a cytoplasmic protein tyrosine phosphatise (Matozaki et al., 2009). Accumulating data indicate that SHP-2 plays important roles in several cell functions (including cell proliferation, survival, metastasis, and so on) induced by cytokines and growth factors (Neel et al., 2003). As a critical mediator of PDGFRs, SHP-2 is recruited to tyrosine residues within the PDGFR cytoplasmic tail after PDGF stimulation and participates in regulating the activation of downstream signaling pathways (Kazlauskas et al., 1993; Rönnstrand et al., 1999). Moreover, previous studies have indicated the crucial effects of SHP-2 on cell growth in retinal cells. The silencing of SHP-2 expression leads to increased apoptosis in retinal cell types (Cai et al., 2011). In TSP1^−/−^ retinal endothelial cells, increased expression of SHP-2 associated with PECAM-1 promotes cell migration and proliferation (Wang et al., 2005). Therefore, we wanted to determine whether SHP-2 participates in PDGF-B-regulated retinal angiogenesis by acting as a mediator.

In the present study, we aimed to determine the roles of PDGF-B in cellular proliferation and angiogenesis in retinal microvascular endothelial cells (RMECs) and to study the corresponding mechanism involved. Our results showed that the cell proliferation and angiogenesis induced by PDGF-B were attenuated by knockdown of either SHP-2 or c-Myc in RMECs. Moreover, PDGF-B positively regulated the activation of c-Myc *via* the SHP-2/Erk pathway in RMECs. These results indicate that PDGF-B facilitates cell proliferation, at least in part, *via* the SHP-2/Erk/c-Myc pathway, which provides potential treatment targets for retinal microangiopathy.

## Materials and Methods

### Materials and Cell Culture

Platelet-derived growth factor-B was purchased from PeproTech (NJ, USA, catalog no. 315-18). Imatinib and U0126 were obtained from Selleck (Shanghai, China, catalog no. S2475 and S1102). We purchased primary RMECs from Cell Biologics Company (Chicago, IL, USA; catalog no. RA-6065). RMECs were cultured in an endothelial cell medium (ScienCell, CA, USA; catalog no. 1001) containing 1% endothelial cell growth supplement (ScienCell, CA, USA, catalog no. 1052) and 10% fetal bovine serum (FBS) in a 5% CO_2_ atmosphere at 37°C. Cells between passages 4 and 8 were utilized in further experiments. Before every experiment, we first starved cells with a serum-free medium for 24 h and then changed the culture medium to a basal medium containing only 1% FBS to minimize the interference of growth factors present in FBS. The control group included cells cultured in a basal medium containing only 1% FBS, while 5 μM imatinib, 20 μM U0126, or exogenous PDGF-BB (20 ng/ml) was added to the indicated groups. The treatment time and sample size for cell counting kit-8 (CCK-8) and bromodeoxyuridine (BrdU) incorporation assays were 48 h and *n* = 6, and the treatment time for other assays was 24 h and *n* = 3.

### Short Interfering RNA Transfection

We purchased the short interfering RNA (siRNA) from GenePharma (Shanghai, China) to knock down the gene expression. The siRNA sequences targeting SHP-2 and Myc were listed as follows: 5'-GGACCAGACAAGUGGCGAU-3' (siSHP-2) and 5'-GAACAUCAUCAUCCAGGAC-3' (siMyc). Transfection of siRNA was performed by using the Lipofectamine 2000 reagent (Invitrogen, CA, USA) according to the manufacturer’s instruction. The knockdown efficiency was verified by Western blot.

### CCK-8 Assay

Cell viability was measured by the CCK-8. Briefly, we cultured the cells into 96-well plates at a density of 3 × 10^3^ cells per well. CCK-8 solution (Dojindo, Kumamoto, Japan; 10 μl) was added to each well, and the plates were incubated at 37°C for 2 h. Then, a microplate reader (Thermo Scientific, Rockford, IL, USA) was utilized to record the absorbance of the samples at a wavelength of 450 nm.

### Measurement of SHP-2 Phosphatase Activity

The phosphatase activity of SHP-2 was examined by using the Active SHP-2 DuoSet IC kit (R&D Systems) according to the manufacturer’s instructions. Briefly, samples were mixed with immunoprecipitation agarose beads, and the mixture was shaken at ~600 rpm and incubated at 4°C for 3 h. After washing with dithiothreitol-containing buffer, we added 10 μl of diluted tyrosine phosphatase substrate I to each reaction, and the mixture was shaken at 600 rpm and 37°C for 30 min. Then, the supernatant was transferred to a 96-well plate, and 10 μl of malachite green reagent A was added to each sample and incubated for 10 min at room temperature. We next added 10 μl of malachite green reagent B into each well and incubated it for 20 min at room temperature. The absorbance was read at 620 nm by a microplate reader (Thermo Scientific).

### BrdU Assay

We detected cell proliferation by using a cell proliferation enzyme-linked immunosorbent assay (ELISA) and a 5-bromo-2'-deoxyuridine (BrdU) kit (Roche Diagnostics; IN, USA). Briefly, 5 × 10^3^ cells per well were seeded in 96-well plates and allowed to attach overnight. Then, we added 10 μl of BrdU labeling solution to each well, and the plates were incubated at 37°C for 24 h. After cell fixation and DNA denaturation with 200 μl of FixDenat, cells were labeled with 200 μl of anti-BrdU-peroxidase solution for 1.5 h at room temperature. We then washed the samples with washing solution [phosphate buffered solution (PBS), 1×] and added 100 μl of tetramethyl-benzidine substrate solution into each well for a 30 min incubation at room temperature. The absorbance was measured at 450 nm by a microplate reader (Thermo Scientific).

### Western Blot

We extracted total proteins by using RIPA buffer (Beyotime, Haimen, China) with protease inhibitor cocktails (Roche Diagnostics) and measured the protein concentration of the lysates with a bicinchoninic acid (BCA) kit (Beyotime). Equal amounts of proteins (40 μg) were separated on 10% sodium dodecyl sulfate-polyacrylamide gel electrophoresis (SDS-PAGE) gels and transferred to polyvinylidene fluoride (PVDF) membranes. After the block in 5% skim milk, the membranes were incubated with the following primary antibodies at 4°C overnight [Proliferating cell nuclear antigen (PCNA), 1:500, Cell Signaling Technology, 2,586; SHP-2, 1:500, Cell Signaling Technology, 3,397; c-Myc, 1:500, Cell Signaling Technology, 18,583; Phospho-c-Myc (Ser62), 1:500, Cell Signaling Technology, 13,748; p-Erk1/2, 1:500, Cell Signaling Technology, 4,695; Erk1/2, 1:500, Cell Signaling Technology, 9,102]. Following washes by PBS-T for 35 min, the membranes were incubated with corresponding secondary antibodies conjugated with horseradish peroxidase for 1 h at room temperature. After washing with PBS-T for 35 min, immunoblots were then developed by a chemiluminescence kit (Pierce ECL, Thermo Scientific). The quantification of the bands was done *via* Image J software.

### Tube Formation Assay

We added 50 μl of growth factor-reduced Matrigel (Becton-Dickinson, MA, USA) into each well of 96-well plates and incubated the plates at 37°C for 30 min. Then, cells were cultured on top of the Matrigel at a density of 1 × 10^4^ cells per well. After a 12 h culture at 37°C, each well was imaged under a phase-contrast microscope (Leica Microsystems, Wetzlar, Hesse-Darmstadt, Germany).

### Cell Migration Assay

The transwell migration assay was used to detect cell migration. A total of 3 × 10^3^ cells were seeded in the top chamber of each insert (BD Biosciences, NJ, USA) with a non-coated membrane. After 12 h of incubation at 37°C, we scraped off the nonmigrated cells in the upper chamber with cotton. Then, the migrated cells were fixed and stained with dye solution (containing 0.1% crystal violet and 20% methanol). Each chamber was imaged under a phase-contrast microscope (Leica Microsystems, Wetzlar, Hesse-Darmstadt, Germany).

### Statistical Analysis

All values were represented as mean ± standard error of the mean (SEM) from at least three independent experiments. Unpaired *t*-test or one-way ANOVA followed by Dunnett’s test was used to evaluate the statistical significance. *p* < 0.05 was considered to be statistically significant.

## Results

### Activation of the PDGF-B/PDGFR Pathway Promotes Cell Proliferation and Angiogenesis

**Figure 1:**
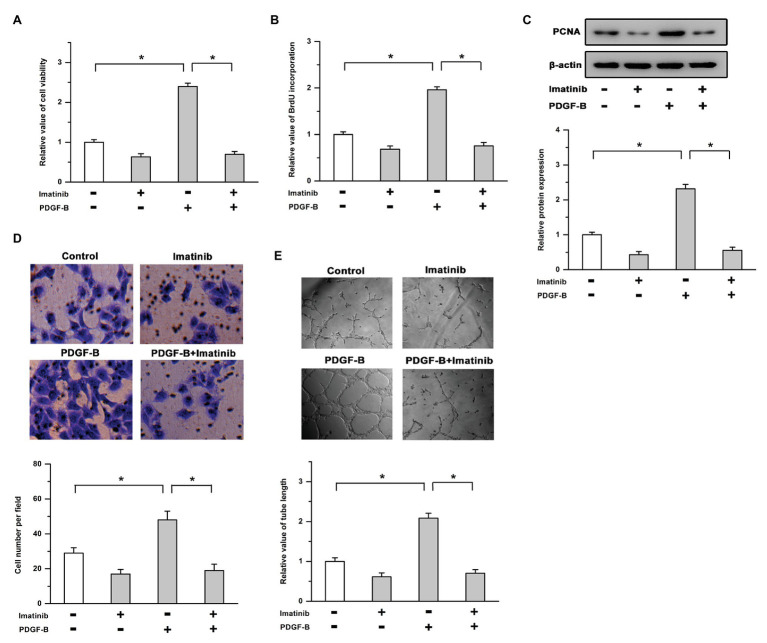
Activation of the platelet-derived growth factor-B/PDGF receptor pathway promotes cell proliferation and angiogenesis. **(A,B)** The increased cell viability **(A)** and bromodeoxyuridine (BrdU) incorporation **(B)** induced by PDGF-B were mitigated by the inhibition of PDGFR. **(C)** PDGF-B-induced proliferating cell nuclear antigen (PCNA) expression was attenuated by imatinib. **(D,E)** Treatment with PDGF-B facilitated cell migration **(D)** and tube formation **(E)** which were mitigated by imatinib. ^*^indicates *p* < 0.05.

To determine the roles of PDGF-B/PDGFR in cell proliferation and angiogenesis, we used exogenous PDGF-B and an inhibitor of PDGFR (imatinib) in this study. Our results showed that treatment with PDGF-B led to increased cell viability, promoted the incorporation of BrdU into newly synthesized DNA, and induced the expression of PCNA in RMECs, which were mitigated by the inhibition of PDGFR (Figures 1A–C). As shown in Figures 1D,E, PDGF-B-facilitated cell migration and tube formation were attenuated by imatinib in RMECs. These results indicate that cell proliferation and angiogenesis are enhanced by the activation of the PDGF-B/PDGFR pathway in RMECs.

### SHP-2 Is Required for PDGF-B-Promoted Cell Proliferation in RMECs

**Figure 2:**
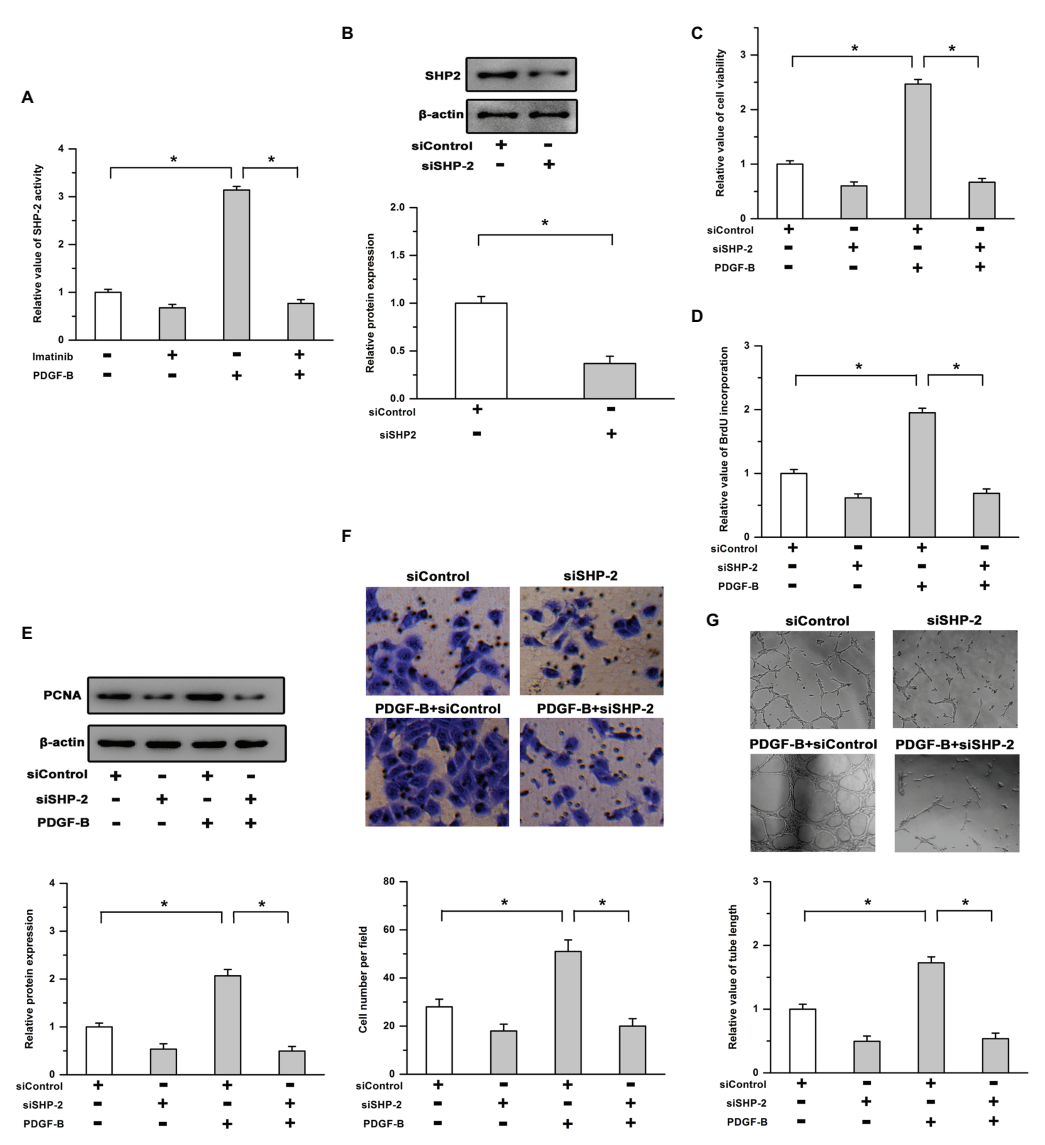
Src homology 2 domain-containing tyrosine phosphatase 2 (SHP-2) is involved in PDGF-B-promoted retinal neovascularization. **(A)** Treatment with PDGF-B led to increased activity of SHP-2, which was attenuated by the inhibition of PDGFR. **(B)** The protein levels of SHP-2 were significantly reduced by SHP-2 siRNA. **(C,D)** PDGF-B increased cell viability **(C)** and promoted BrdU incorporation **(D)** which were reversed by knockdown of SHP-2. **(E)** PDGF-B-induced PCNA expression was mitigated by SHP-2 knockdown. **(F,G)** The cell migration **(F)** and tube formation **(G)** facilitated by PDGF-B stimulation were attenuated by knockdown of SHP-2. ^*^indicates *p* < 0.05.

It has been reported that SHP-2 is an important signaling molecule associated with activated PDGFR and is involved in regulating the survival of all retinal cell types (Cai et al., 2011; Zhang et al., 2016); thus, we examined whether SHP-2 participates in PDGF-B-promoted retinal neovascularization. As shown in Figure 2A, the activity of SHP-2 was significantly increased by PDGF-B stimulation, which was reversed by the inhibition of PDGFR. We also utilized SHP-2 siRNA to repress SHP-2 expression. The efficiency of SHP-2 knockdown was verified by Western blot in RMECs (Figure 2B). Our results showed that the increased cell viability induced by PDGF-B was abrogated by siSHP-2 (Figure 2C). The knockdown of SHP-2 lessened the promoting effects of PDGF-B on BrdU incorporation and PCNA expression (Figures 2D,E). Moreover, the cell migration and tube formation facilitated by PDGF-B stimulation were mitigated by knockdown of SHP-2 in RMECs (Figures 2F,G). These results imply that SHP-2 is involved in PDGF-B-promoted retinal neovascularization by acting as an essential mediator.

### Effects of PDGF-B/SHP-2 in Cell Growth and Angiogenesis Are Mediated by c-Myc

**Figure 3:**
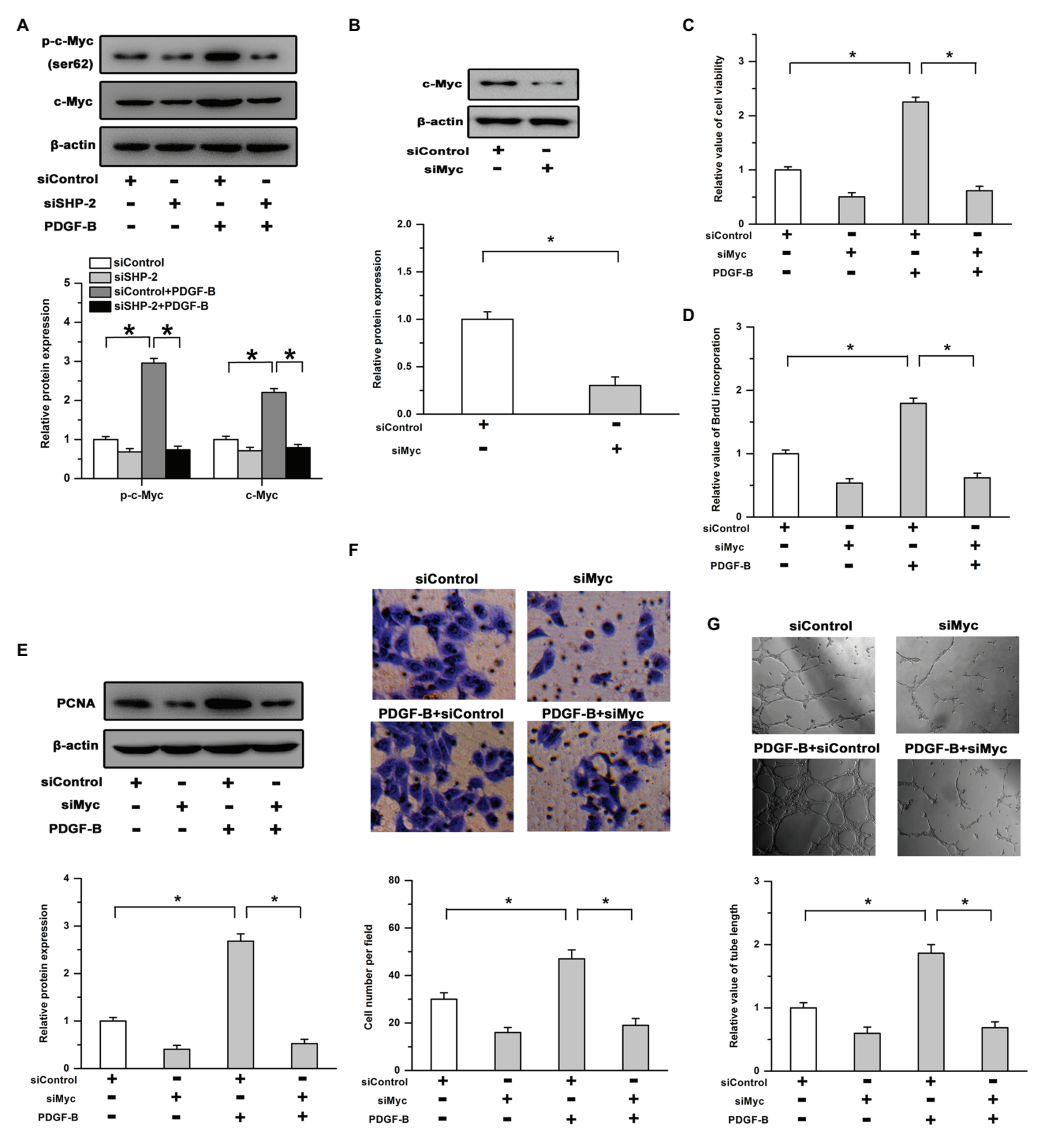
The effects of PDGF-B/SHP-2 on cell growth and angiogenesis are mediated by c-Myc. **(A)** Treatment with PDGF-B induced the phosphorylation and expression of c-Myc, while knockdown of SHP-2 repressed the effects of PDGF-B. **(B)** The expression of c-Myc was significantly depressed by siMyc (an siRNA of c-Myc). **(C,D)** The cell viability **(C)** and BrdU incorporation **(D)** enhancements induced by PDGF-B treatment were attenuated by siMyc. **(E)** Knockdown of c-Myc weakened the promoting effects of PDGF-B on PCNA expression. **(F,G)** PDGF-B-facilitated cell migration **(F)** and tube formation **(G)** were abolished by knockdown of c-Myc. ^*^indicates *p* < 0.05.

Previous studies have shown that the transcription targets of c-Myc are positively regulated by SHP-2 and that c-Myc plays an important role in cell proliferation and angiogenesis as a transcription factor (Aceto et al., 2012; Riddell et al., 2018). The phosphorylation of c-Myc on Ser62 controls its stability and transcriptional activity. Therefore, we examined the relationship between c-Myc and PDGFB/SHP-2 in RMECs. As shown in Figure 3A, treatment with PDGF-B induced the phosphorylation (Ser62) and expression of c-Myc in RMECs, which were mitigated by knockdown of SHP-2. Moreover, an siRNA targeting c-Myc (siMyc) was utilized to depress its expression (Figure 3B). Our results showed that the promoting effects of PDGF-B on cell viability, BrdU incorporation, and PCNA expression were attenuated by siMyc (Figures 3C–E). In addition, PDGF-B-facilitated cell migration and tube formation were weakened by knockdown of c-Myc in RMECs (Figures 3F,G). These results indicate that c-Myc is involved in PDGF-B/SHP-2-regulated cell proliferation and angiogenesis in RMECs.

### The Regulatory Effects of PDGF-B on c-Myc Are Mitigated by the Inhibition of the Erk Pathway

**Figure 4:**
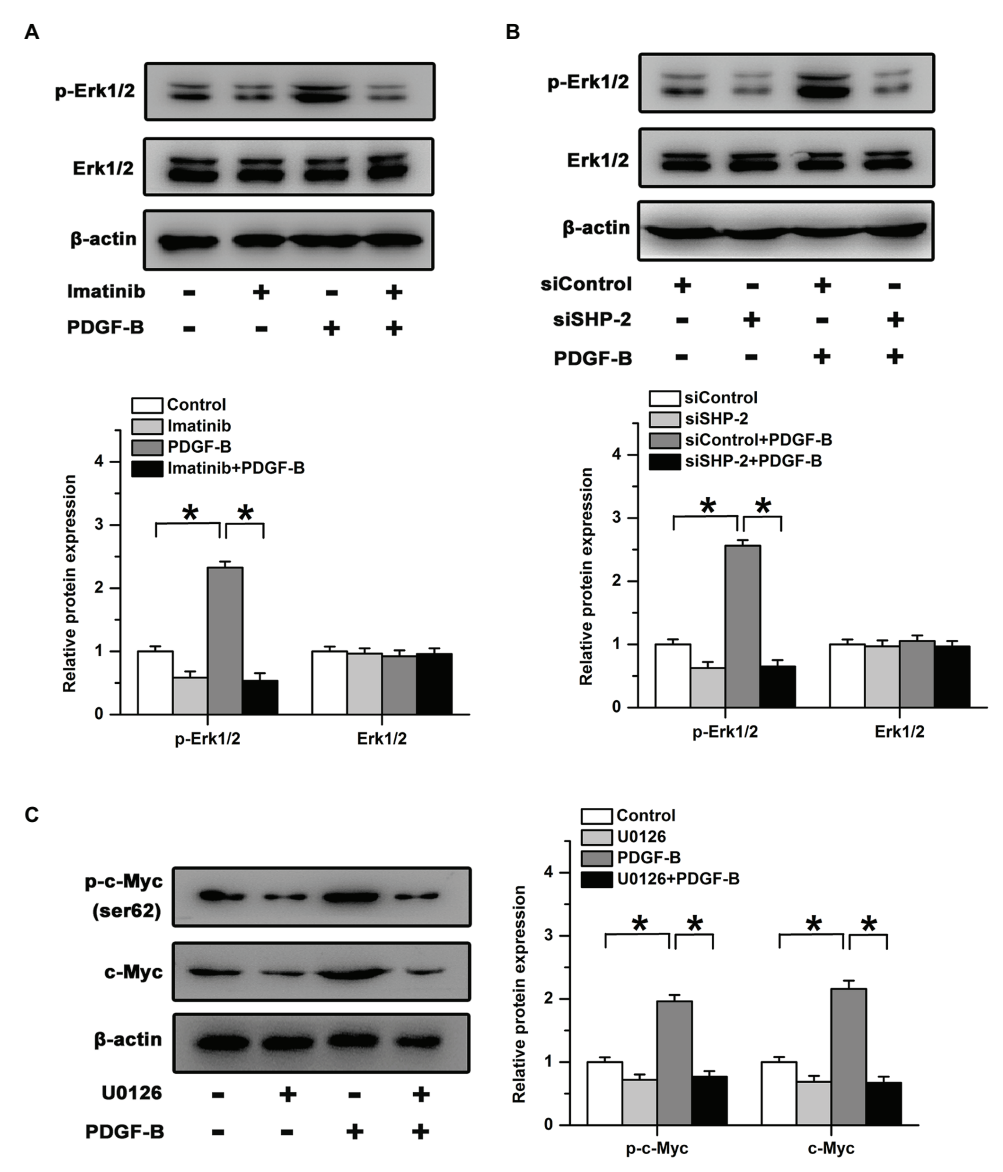
The regulatory effects of PDGF-B on c-Myc expression are mitigated by the inhibition of the Erk pathway. **(A)** Inhibition of PDGFR mitigated the phosphorylation of Erk induced by PDGF-B. **(B)** The phosphorylation of Erk increased by PDGF-B was attenuated by knockdown of SHP-2. **(C)** The increased expression and phosphorylation of c-Myc induced by PDGF-B were mitigated by U0126. ^*^indicates *p* < 0.05.

Src homology 2 domain-containing tyrosine phosphatase 2 preferentially binds to Ras and activates the downstream Erk signaling pathway (Bunda et al., 2015). We examined the activation of Erk after treatment with PDGF-B in RMECs. Our results showed that treatment with PDGF-B induced the phosphorylation of Erk in RMECs, which was blocked by an inhibitor of PDGFR (Figure 4A). Similarly, the increase in Erk phosphorylation induced by PDGF-B was attenuated by knockdown of SHP-2 (Figure 4B). To identify whether the Erk pathway participates in PDGF-B/SHP-2-regulated activation of c-Myc in RMECs, we used U0126 (20 μM) to block the Erk pathway. Our results showed that the expression and phosphorylation of c-Myc induced by PDGF-B were mitigated by U0126 (Figure 4C). The results imply that PDGF-B facilitates the activation of c-Myc *via* the SHP-2/Erk pathway in RMECs.

### Inhibition of the Erk Pathway Attenuated the Effects of PDGF-B on Cell Proliferation and Angiogenesis

**Figure 5:**
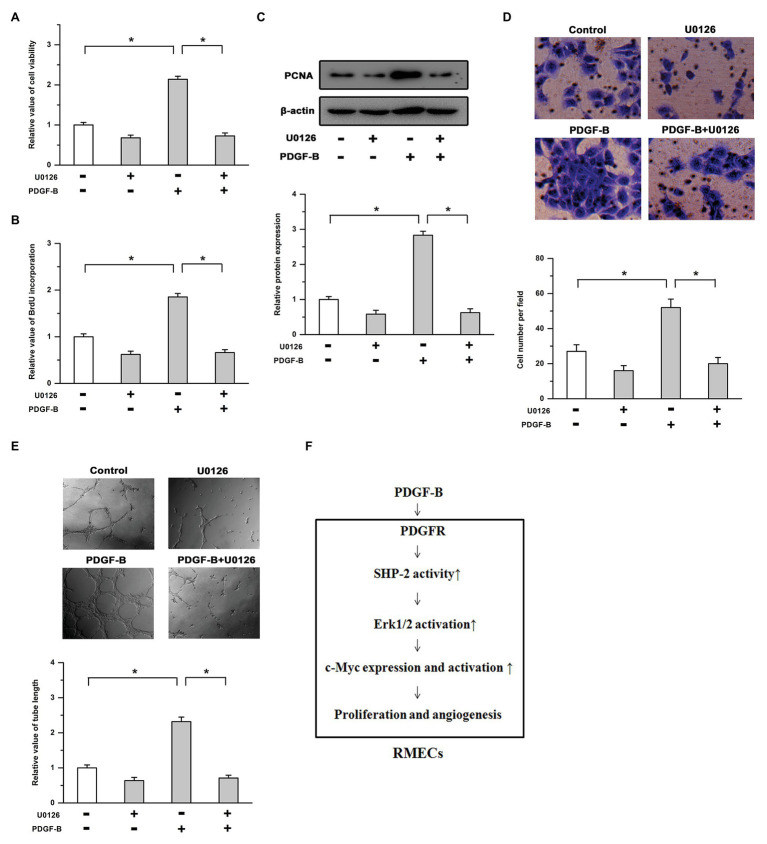
The roles of PDGF-B in cell proliferation and angiogenesis are attenuated by inhibition of the Erk pathway. **(A,B)** The increases in cell viability **(A)** and BrdU incorporation **(B)** induced by PDGF-B were abolished by U0126. **(C)** The increased expression of PCNA induced by PDGF-B was weakened by U0126. **(D,E)** The cell migration **(D)** and tube formation **(E)** enhancements induced by PDGF-B treatment were antagonized by inhibition of the Erk pathway. **(F)** A schematic representation of PDGF-regulated cell proliferation and angiogenesis in retinal microvascular endothelial cells (RMECs) is shown. ^*^indicates *p* < 0.05.

We further studied the roles of the Erk pathway in PDGF-B-regulated cell proliferation in RMECs. Our results showed that the cell viability increase induced by PDGF-B was weakened by U0126 (Figure 5A). PDGF-B promoted BrdU incorporation and PCNA expression, which were mitigated by U0126 (Figures 5B,C). Moreover, the enhanced cell migration and tube formation induced by PDGF-B were antagonized by the inhibition of the Erk pathway (Figures 5D,E). These results imply that PDGF-B promotes cell proliferation and angiogenesis, at least in part, *via* the SHP-2/Erk pathway in RMECs.

## Discussion

Blood vessels, which supply oxygen, nutrients, and metabolites to the retina, play important roles in regulating the normal function and homeostasis of the retina (Selvam et al., 2018). Disruption of the vascular system leads to a decrease in oxygen and nutrient delivery to the retina (Moran et al., 2016). As a consequence, the increases in angiogenic and growth factors induced by retinal ischemia promote blood vessel growth and result in ischemia-induced retinopathies. Although both surgeries and anti-VEGF therapy are therapeutic interventions used in the clinic, visual acuity is significantly weakened by surgery-induced retinal damage, and some patients do not show sensitivity to or benefit from anti-VEGF therapy (Sankar et al., 2016). Therefore, it is necessary to investigate the functions of other critical growth factors involved in modulating retinal neovascularization and explore the regulatory mechanisms. In the present study, our results show that SHP-2-regulated activation of Erk/c-Myc is critical for PDGF-B/PDGFR signaling-facilitated RMEC proliferation and angiogenesis (Figure 5F).

One of the important findings of this study is that PDGF-B-promoted cell proliferation and angiogenesis are mediated by SHP-2. Hypoxia, an important reason for angiogenesis, has been reported to induce the expression of PDGF-B. In proliferative retinal membranes, the expression of PDGF-B and its receptors is significantly upregulated (Robbins et al., 1994). Moreover, the specific expression of PDGF-B in the retina leads to severe neovascularization in mice (Seo et al., 2000). These reports indicate that the PDGF-B/PDGFR pathway is likely to be an important factor in retinal neovascularization. In our study, we found that PDGF-B promoted cell proliferation and enhanced cell migration and tube formation in RMECs, which were attenuated by an inhibitor of PDGFR. SHP-2 is a protein tyrosine phosphatase and has been demonstrated to activate the Ras/MAPK signaling pathway (Scott et al., 2011; Bunda et al., 2015). Previous studies have shown that SHP-2, as a downstream SH2 domain-containing signaling molecule, is activated by residues in the cytoplasmic domain of PDGFR (Ekman et al., 2002; Zhang et al., 2016). Activation of SHP-2 plays an important role in positively regulating HIF-1α stabilization and angiogenesis (Wang et al., 2009; Heun et al., 2017). However, whether SHP-2 participates in PDGF-B-promoted cell proliferation in RMECs has not been clear. Our results showed that treatment with PDGF-B increased the activity of SHP-2 and that the promoting effects of PDGF-B on cell proliferation and angiogenesis were mitigated by knockdown of SHP-2 in RMECs. Furthermore, Erk, as a direct downstream target of SHP-2 (Bunda et al., 2015), was involved in PDGF-B-regulated cell proliferation and angiogenesis. These results imply that activation of SHP-2/Erk is necessary for PDGF-B-facilitated cell proliferation in RMECs.

Another notable finding of this work is that the activation of c-Myc regulated by SHP-2 is required for PDGF-B-promoted cell proliferation and angiogenesis. c-Myc plays critical roles in many cellular processes, such as growth, proliferation, differentiation, and apoptosis, by regulating a substantial number of genes (He et al., 2008; Sun et al., 2015). It has been reported that c-Myc participates in regulating vasculogenesis and angiogenesis during development and tumor progression (Baudino et al., 2002; Cho et al., 2010). Overexpression of c-Myc promotes DNA replication and entry into the S phase (Dominguez-Sola et al., 2007). Moreover, phosphorylation of c-Myc controls its stability. Phosphorylation of c-Myc on Ser62 stabilizes the c-Myc protein and enhances its transcriptional activity (Sears et al., 2000). In our study, we found that treatment with PDGF-B promoted the phosphorylation of Ser62 and the expression of c-Myc. Knockdown of c-Myc expression mitigated the promoting effects of PDGF-B on cell proliferation and angiogenesis. Moreover, the effects of PDGF-B on the expression of c-Myc were significantly attenuated by knockdown of SHP-2 or treatment with U0126. These results imply that PDGF-B promotes cell proliferation and angiogenesis, at least in part, by activating the SHP-2/Erk/c-Myc pathway.

In summary, our results indicate that SHP-2/Erk-facilitated activation of c-Myc plays an important role in cell proliferation and angiogenesis induced by PDGF-B in RMECs. These results might provide new potential targets for the treatment of retinal neovascularization in the future.

## Data Availability Statement

The raw data supporting the conclusions of this article will be made available by the authors, without undue reservation.

## Author Contributions

JM, JW, and GX designed the experiments. WT, LZ, and RG performed the experiments. JM, LZ, and FH analyzed the results. JW and GX prepared the submission. All authors contributed to the article and approved the submitted version.

### Conflict of Interest

The authors declare that the research was conducted in the absence of any commercial or financial relationships that could be construed as a potential conflict of interest.
